# Effects of exercise therapy in patients with pancreatic cancer: A systematic review and meta-analysis

**DOI:** 10.1016/j.ijnsa.2025.100398

**Published:** 2025-08-05

**Authors:** Mizuki Sekino, Takuya Fukushima, Katsuyoshi Suzuki, Keiichi Osaki, Ikumi Yokoyama, Kazuko Katagiri, Shinichiro Morishita, Naoko Sato

**Affiliations:** a School of Nursing, Fukushima Medical University, Fukushima, Japan; b Faculty of Rehabilitation, Kansai Medical University, Osaka, Japan; c Division of Rehabilitation Medicine, Shizuoka Cancer Center, Shizuoka, Japan; d Department of Rehabilitation, Panasonic Health Insurance Organization, Matsushita Memorial Hospital, Osaka, Japan; e Department of Physical Therapy, School of Health Science, Fukushima Medical University, Fukushima, Japan

**Keywords:** Pancreatic cancer, Exercise, Physical function, Quality of life, Meta-analysis

## Abstract

**Introduction:**

The reported benefits of exercise therapy in patients with pancreatic cancer include maintaining and improving physical fitness and muscle strength, reducing treatment-related side effects, and enhancing quality of life. However, the evidence remains inconclusive, necessitating the integration of interventional studies to reach a consensus. This study aimed to elucidate the effects of exercise interventions for patients with pancreatic cancer through a meta-analysis of randomized controlled trials.

**Methods:**

A literature search was conducted to identify articles published prior to May 2024, using the PubMed/MEDLINE, Scopus, CINAHL, and PEDro databases. Search terms included pancreatic cancer, exercise-related terminology, physical function, and quality of life. The primary outcome was quality of life, and the secondary outcome was physical function. All meta-analyses were conducted using a random-effects model.

**Results:**

The final analysis included 6 studies, with sample sizes ranging from 40 to 172 patients. The intervention types were resistance training in 3 studies and a combination of aerobic exercise and resistance training in 3 studies. The timings of the interventions were after surgery or chemotherapy in 4 studies and during chemotherapy or chemoradiotherapy in 2 studies. Three randomized controlled trials used European Organization for Research and Treatment of Cancer Quality of Life Questionnaire-C30 to assess quality of life. Physical function was evaluated using muscle strength measurements (isokinetic and isometric strength), the 5-chair stand test, and the 6-min walk test for exercise capacity. Exercise intervention was effective for improving physical quality of life (standardized mean difference = 0.41, 95 % confidence interval = 0.07–0.74, *p* = 0.02). In addition, improvements in both upper extremity muscle strength (standardized mean difference = 0.50, 95 % confidence interval = 0.21–0.80, *p* = 0.0008) and lower extremity muscle strength were observed (standardized mean difference = 0.35, 95 % confidence interval = 0.14–0.56, *p* = 0.0009). On the other hand, the 6-min walk test showed no significant difference in the effect of exercise therapy between the exercise and control groups.

**Conclusion:**

The findings of this study indicate that exercise therapy for pancreatic cancer patients effectively increases muscle strength in the upper and lower limbs while improving their physical quality of life. However, because all the included studies were assessed as having a high risk of bias, the findings of this review should be interpreted with caution.

**What is already known about this topic**

• Patients with pancreatic cancer often develop cachexia, which leads to a decline in physical function and quality of life.

• While exercise therapy has been shown to improve physical function, muscle strength, and quality of life in some cancers, research into its effects in pancreatic cancer remains limited and inadequate.

• Although systematic reviews have examined the effects of exercise therapy in patients with pancreatic cancer, none has been validated by a meta-analysis.

Alt-text: Unlabelled box

**What this paper adds**

• The present review demonstrated the effects of exercise therapy on improving upper and lower limb muscle strength and physical quality of life in patients with pancreatic cancer.

• To prevent cachexia in patients with pancreatic cancer, it is necessary to accumulate evidence on the effectiveness of exercise therapy through randomized controlled trials and to develop intervention programs that include aerobic exercise.

Alt-text: Unlabelled box

## 1 Introduction

Pancreatic cancer has been increasing year by year, with an age-adjusted incidence rate of 14.5 % in 2020 (Bray et al., 2024). It is difficult to diagnose at an early stage due to the lack of subjective symptoms, and is often detected at an advanced stage when surgical resection is no longer possible. Moreover, it progresses rapidly and has a high potential for metastasis, making it one of the most difficult cancers to treat. Surgery, which is currently the only curative treatment, does not yield a high long-term survival rate on its own, and only about 20–30 % of patients are eligible for surgery with curative intent at the time of diagnosis. Therefore, multidisciplinary treatment combining surgery with neoadjuvant and adjuvant chemotherapy has become the current standard of care, and its effectiveness has been reported. However, the prognosis remains poor, with a 5-year relative survival rate of 8.5 % (Bray et al., 2024) and a high recurrence rate. Treatment is also associated with fatigue, pain, weight loss, muscle weakness, and psychological impairment, which can negatively affect patients’ physical function, including muscle strength and exercise capacity, and health-related quality of life (Maltser et al., 2017). In addition, cachexia commonly occurs in pancreatic cancer, affecting >50 % of patients at the time of diagnosis and 70 % during initial chemotherapy (Mitsunaga et al., 2020). Cachexia also causes the physical dysfunction (Neoptolemos et al., 2017; Conroy et al., 2018). Furthermore, it has become clear that these impairments in physical function and quality of life adversely affect not only activities of daily living, but also survival (Fukushima et al., 2024; Nakano et al., 2021). Therefore, exercise therapy aimed at symptom relief, improving quality of life, and preventing the decline of physical function and activities of daily living is necessary from the early stages of treatment.

Maintaining physical activity is fundamental to sustaining both health and quality of life (Warburton et al., 2006). According to the guidelines from the American Cancer Society (Rock et al., 2022) and the American College of Sports Medicine (Campbell et al., 2019), exercise therapy during the treatment phase improves mental and psychological functions, in addition to physical activity, muscle strength, and physical function. Exercise therapy is recommended as a safe intervention when due consideration is given to the type and stage of cancer, the treatment regimen, and the side effects or complications that may occur as a result. Traditional exercise, such as aerobic and resistance exercise, improves muscle mass, physical function, and physical symptoms during cancer treatment, which may contribute to a reduction in complications associated with both the disease and its treatment (Kamel et al., 2020; Wiskemann et al., 2019; Austin et al., 2024).

However, the clinical studies supporting these recommendations have largely focused on breast cancer, prostate cancer, and blood cancer, and in contrast with the other cancer types, the clinical significance of exercise therapy in pancreatic cancer has not been sufficiently established (Sweegers et al., 2018; Buffart et al., 2017). O'Connor et al. conducted a systematic review of exercise effects and prescription in the treatment of pancreatic ductal adenocarcinoma (O’Connor et al., 2021). However, no meta-analysis was performed due to the lack of randomized controlled trials specific to exercise interventions and their effectiveness in patients with pancreatic cancer, as well as the predominance of observational studies and case reports. In recent years, there have been some studies that have examined the effects of exercise therapy in patients with pancreatic cancer using a randomized controlled design in terms of exercise tolerance, muscle strength, and quality of life (Neuzillet et al., 2023; Weyhe et al., 2022; Ngo-Huang et al., 2023). No meta-analysis has integrated interventional studies to examine the effectiveness of exercise therapy in patients with pancreatic cancer. Several factors may explain the difficulty in conducting long-term interventions and follow-up in this population. These include the high prevalence of cancer cachexia (Mitsunaga et al., 2020), the rapid progression and poor prognosis of the disease (Sheill et al., 2019), and the substantial physical and psychological burden associated with treatment (Luo et al., 2021; Maltser et al., 2017). Due to these challenges, studies targeting pancreatic cancer patients are often limited in scale and duration, making it difficult to accumulate the high-quality data needed for randomized controlled trials and meta-analyses. It is expected that such a meta-analysis would provide novel evidence to guide exercise therapy for pancreatic cancer patients.

In this systematic review, we aimed to clarify the effects of exercise interventions on physical function, muscle strength and quality of life in patients with pancreatic cancer through a meta-analysis of randomized controlled trials.

## 2 Methods

This systematic review was reported in accordance with the Preferred Reporting Items for Systematic Reviews and Meta-Analyses guidelines (Liberati et al., 2009). We used a predefined protocol, and registered it with PROSPERO (CRD42024549311).

### 2.1 Data searches and sources

A literature search was conducted by TF to identify articles published prior to May 2024, using the PubMed/MEDLINE, Scopus, CINAHL, and PEDro databases. Search terms included pancreatic cancer, exercise-related terminology, physical function, and quality of life. The primary outcome was quality of life, and the secondary outcome was physical function. We developed our search strategy in consultation with an experienced medical librarian who specializes in systematic reviews. Detailed search formulas for each database are provided in Supplementary File 1.

### 2.2 Study eligibility criteria and selection

The study eligibility criteria were as follows: 1) randomized controlled trials; 2) original human studies; 3) publications in English; 4) patients with pancreatic cancer in various treatment settings; 5) exercise interventions; and 6) studies evaluating the effects of exercise on physical function, such as muscle strength and exercise capacity, and quality of life. The criteria for exclusion were as follows: 1) systematic reviews, editorials, cross-sectional studies, case reports, case series, and protocols; 2) conference abstracts; and 3) unable to retrieve data such as the mean and standard deviation, even after querying the author. After removing duplicates, 8 reviewers independently assessed the titles and abstracts of all potential studies according to the eligibility criteria. Each reviewer conducted the screening independently to ensure objectivity, and any disagreements were resolved through discussion. Full-text articles were retrieved for review if they were deemed to meet the eligibility criteria or if the abstract and title did not provide sufficient information to make a decision. The final selection of eligible studies was determined in consensus meetings attended by all authors.

### 2.3 Data extraction

The following data were extracted from each included study by 2 reviewers (TF and SM): 1) first author's last name; 2) publication year; 3) nationality; 4) number of patients; 5) age; 6) sex; 7) treatment; 8) intervention; 9) timing of intervention; 10) study duration; 11) control setting; 12) outcome measure; and 13) conclusions. The mean and standard deviation of the post-intervention values were also extracted. No significant differences were assumed between the intervention and control groups at baseline (pre-intervention).

### 2.4 Study quality and risk of bias

Two reviewers (TF and SM) independently evaluated the methodological quality of the included studies using the Cochrane Risk of Bias 2 tool for randomized trials (Sterne et al., 2019). Any disagreements were resolved through consensus meetings attended by all authors. Cochrane Risk of Bias 2 evaluates the following 5 domains of potential bias: bias arising from the randomization process; bias due to deviations from the intended interventions; bias due to missing outcome data; bias in the measurement of the outcome; and bias in the selection of reported results. According to the Cochrane Risk of Bias 2 algorithm, each domain receives a "low risk," "some concerns," or "high risk" rating. An overall risk-of-bias judgment was derived for each study from the domain-level assessments.

### 2.5 Data analysis

Standardized mean differences were calculated with 95 % confidence intervals. Standardized mean differences were considered significant if their 95 % confidence interval did not include zero. All meta-analyses were conducted using a random-effects model. This approach was chosen in consideration of anticipated clinical and methodological heterogeneity across studies, including differences in patient characteristics, intervention protocols, and outcome measures. The use of a random-effects model is consistent with the Cochrane recommendations when heterogeneity is expected. Forest plots were used to visualize the results. Statistical heterogeneity was assessed using the I^2^ statistic, with the I^2^ levels adopted as suggested by the Cochrane Handbook for Systematic Reviews of Interventions (l^2^ values of 0 %, 25 %, 50 %, and 75 % represented no, low, moderate, and high heterogeneity, respectively). The threshold for interpreting the I^2^ value can be misleading. Therefore, the significance of the observed I^2^ value was determined by considering the magnitude and direction of the effect, as well as the strength of evidence for clinical heterogeneity. To improve the interpretability of the results, the muscle strength outcomes were grouped and pooled separately according to region (upper or lower extremity). These groupings were not based on predefined hypotheses regarding subgroup differences; rather, they were intended to provide a clearer summary of the effects of exercise on related outcome measures. All statistical analyses were conducted using Review Manager version 5.1.

## 3 Results

### 3.1 Study selection

**Fig. 1:**
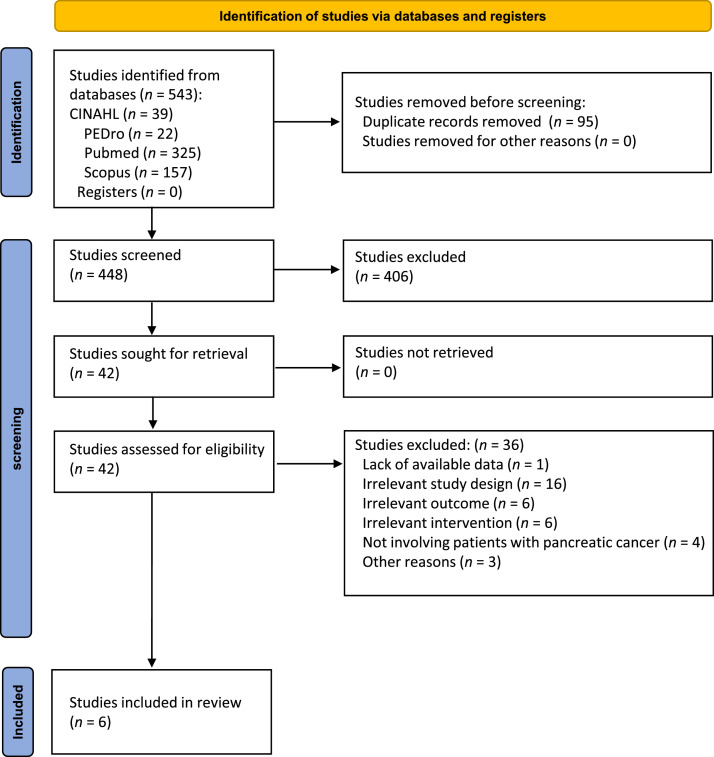
Study flow diagram of the selection process.

A total of 543 articles were retrieved from the database searches, which was reduced to 448 after the removal of duplicate articles. The title and abstract of the 448 studies were screened, and it was found that 406 did not meet the inclusion criteria. Of the remaining 42 full-text publications, 36 studies were excluded for the following reasons: lack of available data (*n* = 1); irrelevant study design (*n* = 16); irrelevant outcomes (*n* = 6); irrelevant interventions (*n* = 6); not involving pancreatic cancer patients (*n* = 4); and other reasons (*n* = 3) (Supplementary File 2). Finally, 6 articles were included in this systematic review and meta-analysis. Fig. 1 provides an overview of the search process and study selection.

### 3.2 Study characteristics

The detailed characteristics and findings of the 6 studies are shown in Table 1 (Kamel et al., 2020; Wiskemann et al., 2019; Neuzillet et al., 2023; Weyhe et al., 2022; Ngo-Huang et al., 2023; Steindorf et al., 2019). The sample sizes of the 6 studies ranged from 40 (Kamel et al., 2020) to 172 patients (Neuzillet et al., 2023). Three studies were conducted in Germany (Wiskemann et al., 2019; Weyhe et al., 2022; Steindorf et al., 2019), while 1 study each was conducted in the USA (Ngo-Huang et al., 2023), France (Neuzillet et al., 2023), and Egypt (Kamel et al., 2020). The intervention types were resistance training in 3 studies (Kamel et al., 2020; Wiskemann et al., 2019; Steindorf et al., 2019) and a combination of aerobic exercise and resistance training in 3 studies (Neuzillet et al., 2023; Weyhe et al., 2022; Ngo-Huang et al., 2023). The timings of the interventions were after surgery or chemotherapy in 4 studies (Kamel et al., 2020; Wiskemann et al., 2019; Weyhe et al., 2022; Steindorf et al., 2019), and during chemotherapy or chemoradiotherapy in 2 studies (Neuzillet et al., 2023; Ngo-Huang et al., 2023). Quality of life was assessed using the European Organization for Research and Treatment of Cancer Quality of Life Questionnaire-C30 in 3 studies (Neuzillet et al., 2023; Weyhe et al., 2022; Steindorf et al., 2019). This instrument assesses global health status/quality of life as the patient’s overall perception of health and quality of life; physical functioning as the ability to perform daily activities; role functioning as the ability to effectively perform various roles in domestic, occupational, and social settings; cognitive functioning as the ability to concentrate and remember; emotional functioning as psychological wellbeing; and social functioning as the impact of illness on family and social life (Aaronson et al., 1993). Physical function was assessed through muscle strength measurements (isokinetic and isometric strength) (Kamel et al., 2020; Wiskemann et al., 2019), the 5-chair stand test (Kamel et al., 2020; Ngo-Huang et al., 2023), and the 6-min walk test for exercise capacity (Wiskemann et al., 2019; Ngo-Huang et al., 2023).

**Table 1:** Characteristics of included studies.

Author Year Country	Patients (number, age, gender)	Treatment	Intervention	Duration	Exercise timing	Measure (Outcome)
Kamel FH 2020 Egypt	Number Total: 40 Exercise group: 20 Control group: 20 Age Total: 51.9 ± 5.0 Exercise group: 51.6 ± 5.2 Control group: 52.3 ± 4.9 Female, 35 %	Surgery, chemotherapy	Resistance exercise - Leg press, leg extension, leg curl, seated row, latissimus pull-down, back extension, butterfly reverse and crunch - First 4 weeks: 50–60 % of 1 repetition maximum From 5 weeks: 60–80 % of 1 repetition maximum - 60 minutes - Twice a week	12 weeks	After surgery or chemotherapy	Maximum isokinetic and isometric peak torque for elbow, knee and hip flexors and extensors, 400-m walk test, 6-m walk test, 5-chair stand test, lean body mass
Neuzillet C 2023 France	Number Total: 172 Exercise group: 87 Control group: 85 Age Total: 64 (29–87) Exercise group: 63 (32–83) Control group: 64 (29–87) Female, 42 %	Chemotherapy	Aerobic exercise - Walking, Nordic walking, or cycling Resistance exercise - Elastic bands	16 weeks	Undergoing first-line chemotherapy	European Organization for Research and Treatment of Cancer Quality of Life Questionnaire-Core 30, nutritional status, insulin resistance, chemotherapy toxicity, progression-free survival, overall survival
Ngo-Huang AT 2023 United States	Number Total: 151 Exercise group: 76 Control group: 75 Age Total: Not reported Exercise group: 66.1 ± 8.5 Control group: 66.2 ± 8.2 Female Total: Not reported Exercise group: 65 % Control group: 57 %	Chemotherapy / chemoradiation	Aerobic exercise - Moderate intensity - ≥30 minutes - ≥ 5 days per week Resistance exercise - 10–15 repetitions of each of eight exercises (major muscle groups) - Body weight and resistance tubes - ≥2 sessions per week	2–6 months	Undergoing neoadjuvant chemotherapy / chemoradiation	Physical activity (modified Godin questionnaire), 6-minute walk distance, 5-chair stand test, handgrip strength, arm curl test repetitions, 3-meter walk test time, Physical functioning (Patient-Reported Outcomes Measurement Information System score), Health-related quality of life (Functional Assessment of Cancer Therapy - Hepatobiliary score), Skeletal muscle index, Skeletal muscle density
Steindorf K 2019 Germany	Number Total: 47 Supervised (Resistance training): 9 Home-based (Resistance training): 21 Control group: 17 Age Total: 60.5 ± 8.4 Supervised (Resistance training): 62.8 ± 6.4 Home-based (Resistance training): 61.0 ± 9.3 Control group: 58.7 ± 8.4 Female, 46.8 %	Surgery, chemotherapy	Supervised resistance training - 8 exercises/session - 2–3 sets with 8–12 repetitions - 60–80 % of 1 repetition maximum - Twice a week - 60 minutes Home-based resistance training - 8 exercises/session - 2–3 sets with 8–12 repetitions - Borg scale: 14–16 - Twice a week - 60 minutes	6 months	At the earliest 3 months after surgical resection	European Organization for Research and Treatment of Cancer Quality of Life Questionnaire-Core 30/ Quality of Life Questionnaire-Pancreatic Cancer Module (26 items), Multidimensional Fatigue Inventory
Weyhe D 2022 Germany	Number Total: 56 Exercise group: 28 Control group: 28 Age Total: 66.4 ± 9.9 Exercise group: 68.0 ± 8.9 Control group: 64.8 ± 10.8 Female, 41.1 %	Surgery	Aerobic and resistance exercise - 3 rounds of in-bed cycling per day (within the first 24 h after extubating) - Walk 3 times/day (15 min each) cycle ergometer muscle exercise with theraband, dumbbells and modified squats 5 days per week (from the second postoperative week) - Encouraged to walk and continue resistance exercise at least 3 times per week (after discharge from the rehabilitation clinic to the 12th postoperative month)	12 months	Long-term immediately after surgery	Short Form-8 Health Survey, European Organization for Research and Treatment of Cancer Quality of Life Questionnaire-Core 30/ Quality of Life Questionnaire-Pancreatic Cancer Module (26 items), Short Physical Performance Battery, survival rate, effects of potential adjuvant therapy on quality of life, and tumor recurrence rate after 6 and 12 months
Wiskemann J 2019 Germany	Number Total: 43 Supervised (Resistance training): 9 Home-based (Resistance training): 20 Control group: 14 Age Supervised (Resistance training): 62.8 ± 6.4 Home-based (Resistance training): 61.*l* ± 8.7 Control group: 58.7 ± 8.2 Female, 44.2 %	Surgery, chemotherapy	Supervised resistance training - Leg press, leg extension, leg curl, seated row, latissimus pull-down, back extension, butterfly reverse, and crunch - First 4 weeks: 50–60 % of 1 repetition maximum From 5 weeks: Borg scale 14–16 - 60 minutes - Twice a week Home-based resistance training - Resistance bands, dumbbells - First 4 weeks: low to moderate intensity From 5 weeks: 60–80 % of 1 repetition maximum - 60 minutes - Twice a week	6 months	At the earliest 3 months after surgical resection	Adherence rate, maximal isokinetic peak torque, maximal voluntary isometric concentration, cardiopulmonary exercise testing, body weight

EORTC QLQ-C30, EORTC Quality of Life Questionnaire-Core 30; FACT, Functional Assessment of Cancer Therapy; MFI, Multidimensional Fatigue Inventory; PROMIS, Patient-Reported Outcomes Measurement Information System; RM, repetition maximum; SPPB, Short Physical Performance Battery.

### 3.3 Risk of bias

**Fig. 2:**
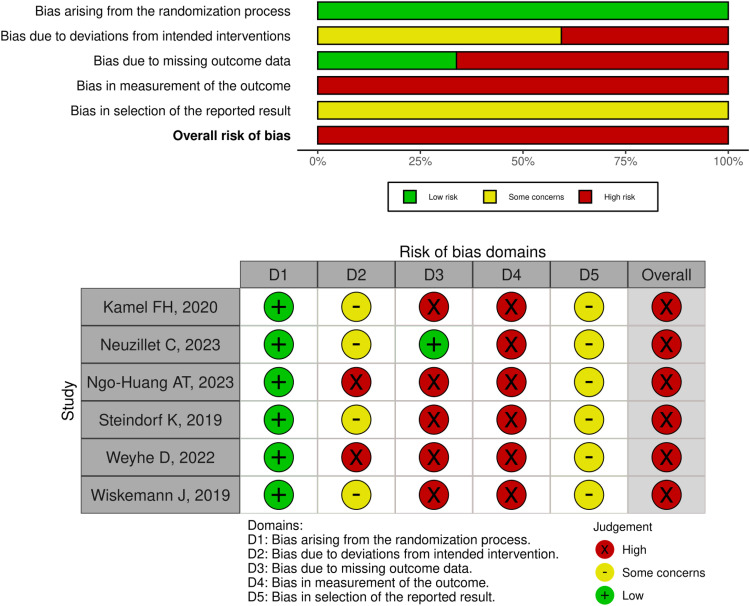
Risk of bias for randomized controlled trials (Cochrane Risk of Bias 2 tool).

The results of the risk of bias assessment are summarized in Fig. 2. All 6 studies included in the analysis were found to have a high risk of bias. The overall high risk of bias across all studies was mainly due to a high risk of bias in outcome measurement, some concerns or high risk of bias related to deviations from intended interventions and selection of reported results, and high risk of bias due to missing outcome data in most studies.

### 3.4 Effect of exercise on quality of life

Three randomized controlled trials investigating exercise's impact on global, physical, role, and cognitive quality of life were analyzed through a random-effects meta-analysis.

**Fig. 3:**
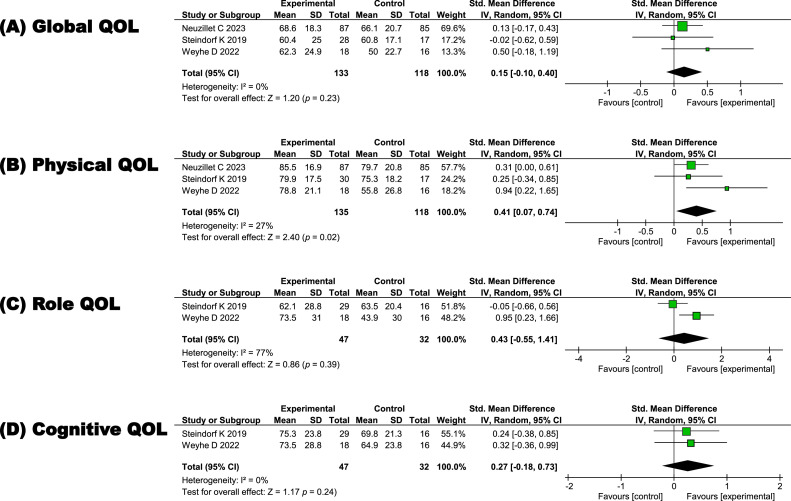
Effect of exercise on quality of life.

The meta-analysis showed a small effect favoring the exercise group over the control group in global quality of life (standardized mean differences = 0.15, 95 % confidence interval = −0.10 to 0.40, I² = 0 %), with considerable uncertainty. For physical quality of life, there was a moderate effect favoring the exercise group (standardized mean differences = 0.41, 95 % confidence interval = 0.07 to 0.74, I² = 27 %), suggesting a meaningful benefit. In the subsequent meta-analysis of 2 randomized controlled trials specifically examining role and cognitive quality of life, small-to-moderate effects favoring the exercise group were observed (role quality of life: standardized mean differences = 0.43, 95 % confidence interval = −0.55 to 1.41, I² = 77 %; cognitive quality of life: standardized mean differences = 0.27, 95 % confidence interval = −0.18 to 0.73, I² = 0 %), though the wide confidence intervals suggest considerable uncertainty (Fig. 3).

### 3.5 Effects of exercise on upper extremity muscle strength

**Fig. 4:**
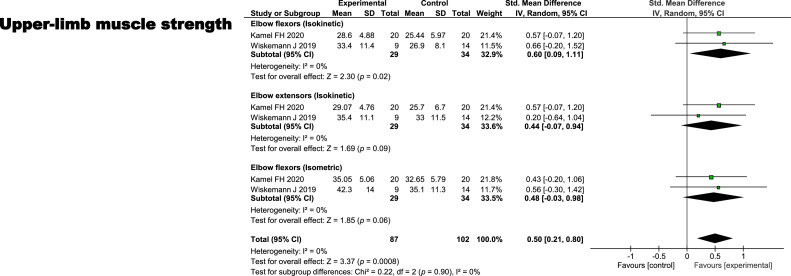
Effect of exercise on upper limb muscle strength.

Two randomized controlled trials investigating the effects of exercise on isokinetic elbow flexor and extensor strength, as well as isometric elbow flexor strength, were included in a random-effects meta-analysis. Overall, upper extremity muscle strength was moderately higher in the exercise group than in the control group (standardized mean differences = 0.50, 95 % confidence interval = 0.21 to 0.80), with no evidence of heterogeneity (I² = 0 %). Isokinetic elbow flexor muscle strength was moderately higher in the exercise group (standardized mean differences = 0.60, 95 % confidence interval = 0.09 to 1.11), suggesting a meaningful benefit with no evidence of heterogeneity (I² = 0 %). The meta-analysis of 2 randomized controlled trials showed small effects favoring the exercise group for both isokinetic elbow extensor muscle strength (standardized mean differences = 0.44, 95 % confidence interval = −0.07 to 0.94, I² = 0 %) and isometric elbow flexor muscle strength (standardized mean differences = 0.48, 95 % confidence interval = −0.03 to 0.98, I² = 0 %), with considerable uncertainty, as indicated by the wide confidence intervals (Fig. 4).

### 3.6 Effect of exercise on lower extremity muscle strength

**Fig. 5:**
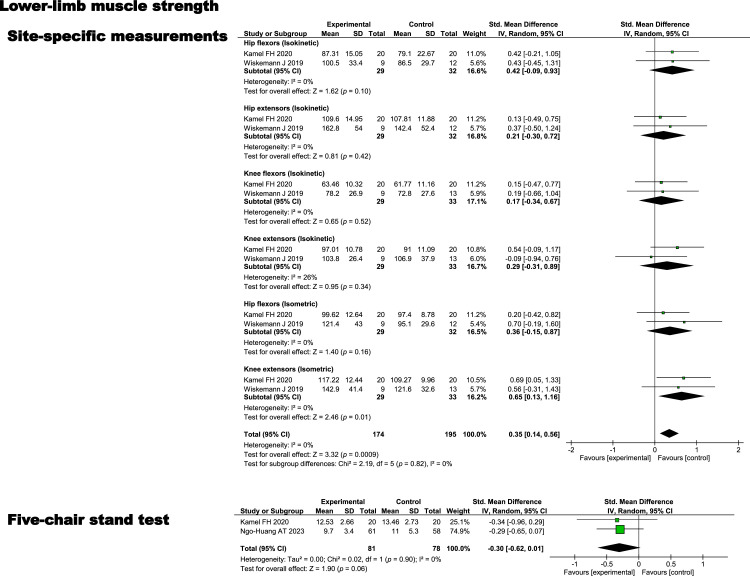
Effect of exercise on lower limb muscle strength.

A random-effects model meta-analysis of the effects of exercise on lower extremity muscle strength included a total of 3 randomized controlled trials. Forest plots were used to present the effects of exercise on isokinetic hip flexor, hip extensor, knee flexor, and knee extensor muscle strength, isometric knee flexor and knee extensor muscle strength, and the 5-chair stand test in patients with pancreatic cancer. Overall, site-specific lower extremity muscle strength was moderately higher in the exercise group than in the control group (standardized mean differences = 0.35, 95 % confidence interval = 0.14 to 0.56), suggesting a small-to-moderate benefit with no evidence of heterogeneity (I² = 0 %). The meta-analysis of 3 randomized controlled trials showed small effects favoring the exercise group for isokinetic hip flexor strength (standardized mean differences = 0.42, 95 % confidence interval = −0.09 to 0.93, I² = 0 %), isokinetic hip extensor strength (standardized mean differences = 0.21, 95 % confidence interval = −0.30 to 0.72, I² = 0 %), isokinetic knee flexor strength (standardized mean differences = 0.17, 95 % confidence interval = −0.34 to 0.67, I² = 0 %), isokinetic knee extensor strength (standardized mean differences = 0.29, 95 % confidence interval = −0.31 to 0.89, I² = 26 %), and isometric hip flexor strength (standardized mean differences = 0.36, 95 % confidence interval = −0.15 to 0.87, I² = 0 %), with considerable uncertainty indicated by wide confidence intervals. Isometric knee extensor muscle strength was moderately higher in the exercise group (standardized mean differences = 0.65, 95 % confidence interval = 0.13 to 1.16, I² = 0 %), suggesting a meaningful benefit. Regarding the 5-chair stand test, no meaningful effect was observed between the exercise and control groups (standardized mean differences = −0.30, 95 % confidence interval = −0.62 to 0.01), suggesting that the intervention had little to no impact on this outcome (I² = 0 %) (Fig. 5).

### 3.7 Effect of exercise on exercise capacity

**Fig. 6:**
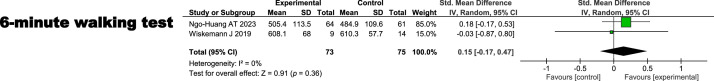
Effect of exercise on exercise capacity.

A random-effects model meta-analysis of 2 randomized controlled trials was conducted to assess the effect of exercise on the 6-min walk test, with the results presented using a forest plot. No meaningful effect was observed between the exercise and control groups (standardized mean differences = 0.15, 95 % confidence interval = −0.17 to 0.47, I² = 0 %), suggesting that the exercise intervention had little to no impact on this outcome (Fig. 6).

## 4 Discussion

The purpose of this study was to elucidate the effects of exercise interventions during the treatment phase of pancreatic cancer on quality of life, muscle strength, and exercise tolerance through a meta-analysis of randomized controlled trials. Six randomized controlled trials met the criteria for inclusion in the analysis. Although the limited number of studies restricts the reliability of the findings, the few discrepancies in the effects of exercise interventions on outcomes suggest that this meta-analysis offers preliminary evidence of the benefits of exercise therapy on quality of life and muscle strength in the upper and lower extremities.

The results of this study showed that exercise interventions were effective in improving upper and lower limb muscle strength. In addition, improvements in upper and lower limb muscle strength were associated with enhanced physical quality of life. On the other hand, improvements in exercise tolerance were not statistically significant.

This study showed that, along with various other cancers (e.g., breast, prostate, and colorectal cancers) for which the muscle-strengthening effects of exercise therapy have been established, there is also a benefit in pancreatic cancer from improved upper and lower limb muscle strength, leading to observed improvements in physical quality of life. Physical function, including muscle strength, has been identified as a factor associated with survival in patients with cancer (Nakano et al., 2021). Considering the high prevalence of cancer cachexia among patients with pancreatic cancer, improvements in muscle strength represent an especially important clinical consideration. The muscle strength gains observed in this meta-analysis suggest that exercise may induce improvements, even under catabolic conditions during treatment. Considering the relationship between muscle strength and survival, improving muscle strength in pancreatic cancer patients could have major clinical significance. More broadly, exercise therapy is expected to be a non-pharmacological intervention for diseases that present with cachexia, including pancreatic cancer. However, a systematic review of exercise interventions for cancer cachexia found that there were no well-designed randomized controlled trials, limited evidence of effectiveness, and no established exercise interventions (Grande et al., 2021). On the other hand, several systematic reviews have shown the efficacy of exercise interventions on physical function, fatigue, and quality of life in patients with advanced cancer, who are at high risk of developing cachexia, although high dropout rates and low adherence to intervention programs have been identified as key challenges (Fayers et al., 2001). Patients with cancer cachexia are often elderly and frail, making them more likely to discontinue exercise therapy due to adverse events during treatments such as chemotherapy and radiation therapy. However, the interventions used in the studies included in this meta-analysis generally involved low- to moderate-intensity exercise performed twice per week over a period of 3–6 months, suggesting that even exercise regimens that are not high in frequency or intensity may still be beneficial (Kamel et al., 2020; Wiskemann et al., 2019). Moreover, the positive effects were observed across a broad range of treatment phases, from the perioperative period to postoperative chemotherapy. Despite concerns regarding adherence in this population, these findings demonstrate that feasible, lower-burden exercise programs can nonetheless lead to improvements in muscle strength. On the other hand, due to the limited number of included studies, it remains difficult to determine the optimal exercise prescription for effectively counteracting muscle loss associated with cancer cachexia. Nonetheless, the present findings suggest that initiating and maintaining exercise from the early phases of treatment may help preserve muscle strength and prevent physical decline, even when the exercise intensity is conservative. Exercise therapy for cancer cachexia still lacks established guidelines regarding optimal frequency, intensity, time, and type. Based on our findings, future research should focus on developing exercise prescriptions tailored to treatment periods and physical symptoms, with the goal of maintaining and improving pancreatic cancer patients’ activities of daily living and instrumental activities of daily living to support their desired quality of life.

In addition, physical quality of life is considered to improve not only through the alleviation of physical symptoms from treatment but also through the enhancement of upper and lower limb muscle strength achieved by exercise interventions. In this meta-analysis, 2 studies (Kamel et al., 2020; Wiskemann et al., 2019) demonstrated the effectiveness of non-pancreatic cancer-specific exercise interventions, performed at low to moderate intensity for 3–6 months, for upper and lower limb muscle strength in pancreatic cancer patients undergoing adjuvant chemotherapy. This suggests that even low-intensity exercise, when initiated early in the treatment phase and maintained, can effectively improve muscle strength associated with activities of daily living, such as basic mobility and standing up. Sato et al. (2015) reported that patients’ perception of being able to do their usual activities 3 months postoperatively was correlated with their physical quality of life. This highlights the significance of early exercise interventions in maintaining and enhancing upper and lower limb muscle strength to prevent disuse syndrome, preserve activities of daily living, and improve quality of life.

In the present study, while physical quality of life was improved, the effect of exercise was not observed in role or cognitive quality of life. Role quality of life was assessed based on a question regarding difficulties in work, hobbies, and leisure activities. Given that patients with pancreatic cancer typically resume work and leisure activities based on improvements in muscle strength, exercise tolerance, and physical symptoms, the timing of the assessment may have influenced the findings of this study. Quality of life reflects patients’ multidimensional perceptions, and its improvement depends on various factors, including physical function, symptom management, and social reintegration. Therefore, healthcare providers need to understand patients’ symptom management abilities and life skills, and a multidisciplinary team should continue providing educational support to enhance patients’ self-management capabilities even after discharge from hospital.

In this study, we could not demonstrate any efficacy of exercise interventions on exercise tolerance. Surgical resection is considered the standard treatment for resectable pancreatic cancer (Doi et al., 2008); however, pancreatic cancer surgery typically involves a wide range of procedures, including those involving the pancreas, stomach, and duodenum, which leads to prolonged postoperative recovery and a high incidence of severe complications that affect recovery (Klaiber et al., 2018; Kawai et al., 2011). In Japan, postoperative chemotherapy is recommended based on reported evidence (Oettle et al., 2013, 2007; Ueno et al., 2009), but the side effects may lead patients to a resting lifestyle, preventing them from participating in active exercise. Presumably, this hinders improvement of exercise tolerance. One of the determinants of exercise tolerance is lower limb muscle strength. In the present review, it was expected that exercise intervention would be effective in improving exercise tolerance, but this was not supported by the findings. Exercise tolerance has been reported to be associated with several factors, including the underlying disease (Allado et al., 2022), treatment-related adverse events (Bai et al., 2025), and physical symptoms (Nakano et al., 2018). However, due to the limited number of randomized controlled trials that were used to examine the effects of exercise therapy in patients with pancreatic cancer, it was not possible to assess these factors at a consistent level. In addition, analyses that included exercise tolerance as an outcome also included a report of resistance training only (Kamel et al., 2020); therefore, future interventions that include aerobic exercise may be needed to improve exercise tolerance.

Palliative rehabilitation for life-threatening illnesses such as cancer, heart failure, and nonmalignant respiratory diagnoses is associated with improvements in quality of life and reduced the length of stay for readmission days (Pryde et al., 2024). This suggests that rehabilitation may be an important form of supportive care for patients with advanced cancer. However, implementing and sustaining exercise interventions and follow-up in populations with advanced cancers characterized by high mortality rates remains a significant challenge. This difficulty stems from the rapid disease progression, poor prognosis, and high prevalence of cancer cachexia commonly observed in these patients. These factors adversely affect recruitment and adherence to exercise programs. In a systematic review, Sheill et al. (2019) identified several barriers to participation in exercise interventions among patients with advanced cancer, including disease progression, time constraints, and transportation difficulties. LeBlanc et al. (2015) further demonstrated that patients with advanced non-small cell lung cancer experience a markedly accelerated decline in their physical function during the course of cancer treatment, particularly in the presence of cachexia. Moreover, previous studies (Luo et al., 2021; Maltser et al., 2017) highlighted that treatment-related physical burdens (e.g., pain, fatigue, anorexia, and dyspnea) can significantly reduce patients’ capacity and willingness to engage in physical activity. These factors may contribute to psychological resistance toward exercise participation. Given the above considerations, the implementation and continuation of exercise interventions in patients with advanced cancer is inherently complex and necessitates ongoing support to ensure adherence and sustain therapeutic effects. Accordingly, future intervention programs must be designed with careful attention to safety and individualized needs, taking into account cancer type, disease stage, and the severity of cachexia. Additionally, addressing system-level challenges, such as enhancing patient motivation, expanding supportive resources, securing adequate healthcare personnel, and developing structured educational and interdisciplinary collaboration frameworks, will be essential for the successful integration of exercise therapy in this vulnerable population.

### 4.1 Study limitations and strengths

One limitation of this study was that some of the included studies did not report the effects of exercise therapy on each outcome. Due to the limited number of studies included in each meta-analysis, formal assessments of publication bias (e.g., funnel plots) and sensitivity analyses were not conducted, as these methods are generally considered unreliable when the number of included studies is small. All of the included studies had a high risk of bias across multiple domains, which, along with the small number of studies and methodological heterogeneity, limits the certainty of the evidence. Therefore, although a meta-analysis was feasible in this review, the findings should be interpreted with caution. A formal Grading of Recommendations, Assessment, Development, and Evaluations assessment was not conducted, as the high risk of bias and small sample size would likely have led to uniformly low certainty ratings. Although mean differences are generally applied in meta-analyses, this was not possible here due to a lack of data. Therefore, post-intervention values were used in the analysis. However, we confirmed that there was no significant variation in the baseline data, and we took this into consideration to avoid affecting the results of the study. Because only a few studies were included, most meta-analyses only included a small number of studies. We did not perform stratification by intervention type, such as aerobic exercise, resistance training, or both. Furthermore, we did not perform sub-analyses, including analyses of the frequency, intensity, and duration of exercise. Moreover, the included studies involved different care models, such as perioperative care and chemotherapy, which were not analyzed separately. Although muscle strength may improve with exercise regardless of the treatment phase, and such improvement could contribute to enhanced physical quality of life, the roles and expectations placed on patients may differ depending on the timing of treatment. This difference in patient experience might account for the absence of significant differences in quality of life scales other than physical quality of life.

This study has several strengths that enhance the reliability and clinical relevance of its findings regarding the effects of exercise therapy in patients with pancreatic cancer. By conducting a meta-analysis of multiple randomized controlled trials, the study reduces individual study biases and yields more generalizable conclusions. The included exercise interventions were diverse, encompassing aerobic exercise, resistance training, and combined modalities, thereby allowing for comparative evaluation of their respective effects. Moreover, the inclusion of patients across different treatment stages—after surgery and during or after chemotherapy—further strengthens the external validity of the findings. Physical function was assessed using objective measures, while quality of life was evaluated using validated questionnaires, enabling a comprehensive assessment of the impact of the interventions. Additionally, the review considers the timing of exercise relative to cancer treatment, providing valuable insights for clinical implementation and future research directions. Future studies should accumulate evidence on the effectiveness of exercise therapy for preventing cachexia in patients with pancreatic cancer, and develop exercise prescriptions tailored to treatment stages and physical symptoms. In addition, to promote the acceptance and continuation of exercise interventions by patients, it is necessary to establish an interdisciplinary team approach.

## 5 Conclusion

The present review demonstrated the effects of exercise therapy on improving upper and lower limb muscle strength and enhancing physical quality of life in patients with pancreatic cancer. In the future, to prevent cachexia in pancreatic cancer patients, it is necessary to accumulate evidence on the effectiveness of exercise therapy through randomized controlled trials, and to develop intervention programs that include aerobic exercise.

## Funding

This study was supported by JSPS KAKENHI grant number 24K02735.

## Data availability statement

The data that support the findings of this study are available from the corresponding author upon reasonable request.

## CRediT authorship contribution statement

**Mizuki Sekino:** Writing – original draft, Investigation, Conceptualization. **Takuya Fukushima:** Writing – review & editing, Visualization, Methodology, Investigation, Formal analysis, Data curation, Conceptualization. **Katsuyoshi Suzuki:** Investigation. **Keiichi Osaki:** Investigation. **Ikumi Yokoyama:** Investigation. **Kazuko Katagiri:** Investigation. **Shinichiro Morishita:** Writing – review & editing, Validation, Supervision, Investigation, Conceptualization. **Naoko Sato:** Writing – review & editing, Supervision, Project administration, Investigation, Funding acquisition, Conceptualization.

## Declaration of competing interest

The authors declare that they have no known competing financial interests or personal relationships that could have appeared to influence the work reported in this paper.

## Appendix Supplementary materials

Image, application 1

Image, application 2
