# Supplementary material for: Effects of exercise therapy in patients with pancreatic cancer: A systematic review and meta-analysis
Source: Int J Nurs Stud Adv. 2025 Aug 5;9:100398. doi: 10.1016/j.ijnsa.2025.100398 (PMC12355156; doi:10.1016/j.ijnsa.2025.100398)
Supplement: Supplementary file 1 [file mmc1.docx]

**Supplementary File 1**

**Search strategy**

PubMed/MEDLINE

| #1 | pancreatic neoplasms[MH] OR pancreatic cancer*[TIAB] OR pancreatic adenocarcinoma*[TIAB] OR pancreatic ductal adenocarcinoma*[TIAB] | 115,173 |
| --- | --- | --- |
| #2 | Physical Therapy Modalities[MH] OR exercise training*[TIAB] OR Exercise[MH] OR aerobic exercis*[TIAB] OR aerobic training*[TIAB] OR resistance training*[TIAB] OR rehabilitation[MH] OR physical activit*[TIAB] OR physical therap*[TIAB] | 705,763 |
| #3 | #1 AND #2 | 350 |
| #4 | #3 AND English[LA] | 325 |

Scopus

| ( TITLE-ABS-KEY ( "pancreatic neoplasm*" OR "pancreatic cancer*" OR "pancreatic adenocarcinoma*" OR "pancreatic ductal adenocarcinoma*" ) ) AND ( TITLE-ABS-KEY ( "exercise therap*" OR "exercise training*" OR exercise* OR "aerobic exercis*" OR "aerobic training*" OR "resistance training*" OR "physical activit*" OR "rehabilitation*" OR "physical therap*" ) ) AND ( TITLE-ABS-KEY ( "physical function*" OR "muscle strength*" OR "Exercise Tolerance*" OR "Exercise Test*" OR gait* OR "Walk Test*" OR "walking test*" OR "muscle mass*" OR "Postural Balance*" OR "exercise capacit*" OR "short physical performance batter*" OR "quality of life" OR "QLQ C30" OR "Functional Assessment of Cancer Therapy-General" OR "FACT G" OR "short form 36" OR "SF 36" ) ) | 157 |
| --- | --- |

CINAHL

| S1 | MH "Pancreatic Neoplasms+" OR pancreatic cancer* OR pancreatic adenocarcinoma* OR pancreatic ductal adenocarcinoma* | 18,811 |
| --- | --- | --- |
| S2 | MH "Physical Therapy+" OR exercise training* OR MH "Exercise+" OR aerobic exercis* OR aerobic training* OR resistance training* OR MH "Rehabilitation+" OR MH "Physical Activity+" OR physical activit* OR physical therap* | 529,916 |
| S3 | physical function* OR MH "Muscle Strength+" OR MH "Exercise Tolerance+" OR MH "Exercise Test+" OR MH "Gait+" OR Walk Test* OR walking test* OR muscle mass* OR MH "Balance, Postural+" OR exercise capacit* OR short physical performance batter* OR MH "Quality of Life+" OR QLQ C30 OR Functional Assessment of Cancer Therapy-General OR FACT G OR short form 36 OR SF 36 | 271,358 |
| S4 | S1 AND S2 AND S3 | 43 |
| S5 | S4 AND LA English | 39 |

PEDro

| Abstract & Title: | pancrea* | 22 |
| --- | --- | --- |
| Subdiscipline: | oncology |  |
