# Supplementary material for: Effects of exercise therapy in patients with pancreatic cancer: A systematic review and meta-analysis
Source: Int J Nurs Stud Adv. 2025 Aug 5;9:100398. doi: 10.1016/j.ijnsa.2025.100398 (PMC12355156; doi:10.1016/j.ijnsa.2025.100398)
Supplement: Supplementary file 2 [file mmc2.docx]

**Supplementary File 2**

**Papers excluded from secondary screening**

| Author | Title | Journal information | Reasons for exclusion |
| --- | --- | --- | --- |
| Ausania F | Prehabilitation in patients undergoing pancreaticoduodenectomy: a randomized controlled trial | Rev Esp Enferm Dig. 2019;111(8):603-608 | Irrelevant outcomes |
| Avancini A | Exploring the feasibility of a combined exercise program for patients with advanced lung or pancreatic cancer | Asia Pac J Oncol Nurs. 2023;10(Suppl 1):100298 | Not involving pancreatic cancer patients |
| Boehm K | Effects of yoga interventions on fatigue: a meta-analysis | Evid Based Complement Alternat Med. 2012;2012:124703. | Irrelevant interventions |
| Brown M | Feasibility of delivering supervised exercise training following surgical resection and during adjuvant chemotherapy for pancreatic ductal adenocarcinoma (PRECISE): a case series | BMC Sports Sci Med Rehabil. 2023;15(1):116 | Irrelevant study design |
| Bundred JR | Prehabilitation prior to surgery for pancreatic cancer: A systematic review | Pancreatology. 2020;20(6):1243-1250 | Irrelevant study design |
| Burgess LC | What is the role of post-operative physiotherapy in general surgical enhanced recovery after surgery pathways? | European Journal of Physiotherapy. 2018;21(2):67–72 | Irrelevant study design |
| Chandrabalan VV | Pre-operative cardiopulmonary exercise testing predicts adverse post-operative events and non-progression to adjuvant therapy after major pancreatic surgery | HPB (Oxford). 2013;15(11):899-907 | Irrelevant interventions |
| Chu P | Effects of accelerated rehabilitation surgery on the clinical prognosis of patients undergoing pancreaticoduodenectomy for pancreatic cancer | Minerva Gastroenterol (Torino). 2022;68(2):253-255 | Irrelevant outcomes |
| Dagorno C | Prehabilitation in hepato-pancreato-biliary surgery: a systematic review and meta-analysis. A necessary step forward evidence-based sample size calculation for future trials | J Visc Surg. 2022;159(5):362-372 | Irrelevant study design |
| De Luca R | Immunonutrition and prehabilitation in pancreatic cancer surgery: A new concept in the era of ERAS® and neoadjuvant treatment | Eur J Surg Oncol. 2023;49(3):542-549 | Irrelevant study design |
| Dong Z | Effectiveness of a multidisciplinary comprehensive intervention model based on the hospital elderly life program to prevent delirium in patients with severe acute pancreatitis | Ann Palliat Med. 2020;9(4):2221-2228 | Not involving pancreatic cancer patients |
| Fagevik Olsén M | Short-term effects of mobilization on oxygenation in patients after open surgery for pancreatic cancer: a randomized controlled trial | BMC Surg. 2021;21(1):185 | Irrelevant interventions |
| Grande AJ | Exercise for cancer cachexia in adults | Cochrane Database Syst Rev. 2021;3(3):CD010804 | Irrelevant study design |
| Heislein DM | Effect of exercise on quality of life and functional performance for patients undergoing treatment for gastrointestinal cancer. | Rehabilitation Oncology. 2009;27(1):3-8 | Not involving pancreatic cancer patients |
| Katsourakis A | How Exercise Can Influence Oxidative Stress and Glucose Levels after Pancreatic Resection: A Randomised Controlled Trial | Dig Surg. 2020;37(3):205-210 | Irrelevant outcomes |
| Kumar R | Cardiopulmonary exercise testing in hepato-biliary & pancreas cancer surgery - A systematic review: Are we any further than walking up a flight of stairs? | nt J Surg. 2018;52:201-207 | Irrelevant interventions |
| Kurokawa H | Changes in physical function and effects on qol in patients after pancreatic cancer surgery | Healthcare (Basel). 2021;9(7):882 | Others |
| Law B | Best supportive care in advanced pancreas cancer: a systematic review to define a patient-care bundle | ANZ J Surg. 2024;94(7-8):1254-1259 | Irrelevant study design |
| Luo H | Feasibility and efficacy of a multicomponent exercise medicine programme in patients with pancreatic cancer undergoing neoadjuvant therapy (the EXPAN trial): study protocol of a dual-centre, two-armed phase I randomised controlled trial | BMJ Open Gastroenterol. 2021;8(1):e000642 | Irrelevant study design |
| Luo H | Evaluation of a Clinic-Based Exercise Program in Patients with Pancreatic Cancer Undergoing Nonsurgical Treatment | Med Sci Sports Exerc. 2023;55(1):9-19 | Irrelevant study design |
| Mikkelsen MK | Effects of a 12-week multimodal exercise intervention among older patients with advanced cancer: results from a randomized controlled trial [with consumer summary] | Oncologist. 2022;27(1):67-78 | Irrelevant interventions |
| Nagamori M | Laparoscopic distal pancreatectomy for pancreatic tail cancer in a 100-year-old patient | Clin J Gastroenterol. 2023;16(5):779-784 | Irrelevant study design |
| Ngo-Huang A | Home-based exercise during preoperative therapy for pancreatic cancer | Langenbecks Arch Surg. 2017;402(8):1175-1185 | Irrelevant study design |
| No author listed | Combination therapy may improve survival and outcome for pancreatic cancer patients | Expert Rev Pharmacoecon Outcomes Res. 2014;5(3):233-234. | Irrelevant interventions |
| O’Neill L | Physical function in patients with resectable cancer of the pancreas and liver–a systematic review | J Cancer Surviv. 2020;14(4):527-544 | Irrelevant study design |
| Okada KI | Supervised Exercise Therapy and Adjuvant Chemotherapy for Pancreatic Cancer: A Prospective, Single-Arm, Phase II Open-Label, Nonrandomized, Historically Controlled Study | J Am Coll Surg. 2022;235(6):848-858 | Irrelevant outcomes |
| Pal A | Resistance Exercise Modulates Kynurenine Pathway in Pancreatic Cancer Patients | Int J Sports Med. 2021;42(1):33-40 | Irrelevant outcomes |
| Parker NH | The Role of Home-Based Exercise in Maintaining Skeletal Muscle During Preoperative Pancreatic Cancer Treatment | Integr Cancer Ther. 2021;20:1534735420986615 | Irrelevant study design |
| Rosebrock K | Effects of Exercise Training on Patient-Specific Outcomes in Pancreatic Cancer Patients: A Scoping Review. | Cancers (Basel). 2023;15(24):5899 | Irrelevant study design |
| Shen Y | Effect of painkillers combined with cognitive intervention on quality of life of postoperative pancreatic cancer patients | Journal of Practical Oncology. 2017;32(3):257-259 | Others |
| Shun SC | Proposing a Comprehensive Prehabilitation Model for Individuals with Operable Pancreatic Cancer. | Asia Pac J Oncol Nurs. 2020;7(3):255-258 | Irrelevant study design |
| Solheim TS | A randomized phase II feasibility trial of a multimodal intervention for the management of cachexia in lung and pancreatic cancer | J Cachexia Sarcopenia Muscle. 2017;8(5):778-788 | Irrelevant study design |
| Steffens D | Individualised, targeted step count intervention following gastrointestinal cancer surgery: The Fit-4-Home randomised clinical trial | ANZ J Surg. 2022;92(4):703-711 | Not involving pancreatic cancer patients |
| Weyhe D | Effects of intensive physiotherapy on Quality of Life (QoL) after pancreatic cancer resection: a randomized controlled trial | MC Cancer. 2022;22(1):520. | Others |
| Wochner R | Impact of progressive resistance training on CT quantified muscle and adipose tissue compartments in pancreatic cancer patients | PLoS One. 2020;15(11):e0242785 | Irrelevant outcomes |
| Yeo TP | A progressive postresection walking program significantly improves fatigue and health-related quality of life in pancreas and periampullary cancer patients | J Am Coll Surg. 2012;214(4):463-75; discussion 475-7 | Lack of available data |
